# Systematic Assessment of *Mycobacterium avium* Subspecies *Paratuberculosis* Infections from 1911–2019: A Growth Analysis of Association with Human Autoimmune Diseases

**DOI:** 10.3390/microorganisms8081212

**Published:** 2020-08-10

**Authors:** Temitope C. Ekundayo, Anthony I. Okoh

**Affiliations:** 1 SAMRC Microbial Water Quality Monitoring Centre, University of Fort Hare, Alice 5700, South Africa; AOkoh@ufh.ac.za; 2 Applied and Environmental Microbiology Research Group, Department of Biochemistry and Microbiology, University of Fort Hare, Alice 5700, South Africa; 3 Department of Biological Sciences, University of Medical Sciences, Ondo City PMB 536, Nigeria

**Keywords:** *Mycobacterium avium* subsp. *paratuberculosis*, MAP, Johne’s disease, Type 1 diabetes mellitus, multiple sclerosis, sarcoidosis, thyroiditis disorders, psoriasis, ulcerative colitis, Parkinson’s disease, arthritis, Blau syndrome, rheumatoid arthritis, resistance, osteopontins

## Abstract

*Mycobacterium avium* subsp. *paratuberculosis* (MAP) is an understudied pathogen worldwide with continuous implications in human autoimmune diseases (ADs). The awareness of MAP appears to be low in many places and its research is at infant stage in many countries. The lack of worldwide coverage of the MAP research landscape calls for urgent research attention and prioritization. This present study aimed to assess MAP global research productivity with an emphasis on its implications in ADs via bibliometric and growth analytic frameworks from authors, countries, institutions, international, disciplines and collaboration network perspectives. MAP primary articles were retrieved from the Scopus database and the Web of Science from 1911 to 2019 via title-specific algorithm. Analytic results of dataset yielded a total of 3889 articles from 581 journals and 20.65 average citations per documents. The annual growth rate of MAP research for the period was 6.31%. Based on a country’s productivity (articles (%), freq. of publication (%)), the USA (887 (22.81%), 26.72%), and Australia (236 (6.07%), 6.07%) ranked the top 2 countries but Egypt and Germany had the highest average growth rate (AGR, 170%) in the last 3 years. MAP studies are generally limited to Europe, Australia, Asia, South America and few nations in Africa. It had positive growth rate (30%–100%) in relation to type 1 diabetes mellitus and rheumatoid arthritis ADs; food science and technology, immunology, agriculture, pathology, and research and experimental medicine, wildlife, environments, virulence, disease resistance, meat and meat products, osteopontin, waste milk and slurry/sludge digestion subjects; but negative growth (−130% to −30%) in ulcerative colitis and Parkinson’s disease and no growth in multiple sclerosis, sarcoidosis, thyroid disorders, psoriasis, and lupus. The mapping revealed a gross lack of collaboration networking in terms of authorship, (intra- and inter-) nationally and institutionally with a generalized collaboration index of 1.82. In conclusion, inadequate resources-, knowledge- and scientific-networking hampered growth and awareness of MAP research globally. The study recommends further research to strengthen evidence of MAP’s epidemiologic prevalence in ADs and proffer practical solution(s) for drug development and point-of-care diagnostics amongst other extended themes.

## 1. Introduction

*Mycobacterium avium* subsp. *paratuberculosis* (MAP) is a known veterinary pathogen of ruminants such as goats, sheep, cattle and other wildlife [1]. It is the aetiological agent of the contagious chronic granulomatous enteritis called Johne’s disease (JD) in ruminants with intramacrophagal survivability, proliferation and dissemination potentials [2]. A recent study isolated MAP from intestines and intestinal lymph nodes and highlighted the role of non-domestic cats reared under local conditions in Egypt in the epidemiology of Johne’s disease outbreak in a mixed camel–cattle farm [3]. Furthermore, MAP has been isolated in non-ruminant animals such as rats, foxes, boars, and weasels [4], highlighting the subject of extended host range. Massive economic losses and cost in terms of animal welfare concerns and production losses have been incurred due to MAP/JD disease in the ruminant/livestock industry worldwide [5,6] to an estimated annual loss between $200 million and $1.5 billion in the cattle industry in the USA alone [7].

Aside from animal infections, MAP is responsible for soft-tissue and lymph-node infections in healthy humans and has been associated with Crohn’s disease and other autoimmune diseases in human as well [8,9,10,11,12]. The ease of faecal-oral dissemination of the pathogen through a number of ways such as feeding, milk/milk products, meat products, direct contact with infected animals, animal faeces/manure, water, and pasture [13,14] and abundance/persistent in the environment [15,16,17], constitute a greater public health risk and menace in general control of MAP; and thus, a greater threat in pre-urban, pastoral and rural communities. As these communities lack potable water supply and get their supplied from pastoral- activities impacted freshwater, which positioned them to contract MAP in a myriad of ways. In addition, agricultural application of solid or liquid digestates of anaerobic processes such as biogas plants loaded with slurry and animal faeces from MAP-infected dairy farms pose further risks of spreading infection. The pathogen has been shown to survive the operational conditions of biogas plants [18]. Also, recent isolation of MAP from free-living environmental amoebae (FLEA) such as the poorly described *Rosculus* genus called for concern on environmental health as FLEA are protozoa found in both aquatic and soil milieus, not only harbour the pathogen but are also capable of intracellular propagation of MAP [19,20]). The outstanding danger of MAP to human public health lies in its parallel health importance with *M. tuberculosis* infections since both infections are characterised by a lengthy latent periods/subclinical cases, multiple disease presentations [1,21], primary macrophage-targeted colonization and possible co-infections in an individual [1].

MAP remains one of the emerging foodborne and zoonotic pathogens in the world that lack detailed critical inspection/studies in humans, freshwater, and dairy industries. Regardless of its worldwide distribution [13,14] and availability of a number of some studies on the pathogen, the cognisance of the pathogen especially in the low-resource countries is very low and limited in geographical coverage. The research response towards this contagious microorganism is still not widespread in many countries due to lack of technical know-how and necessary diagnostic equipment despite its infectious and high-risk tendencies. Therefore, for this aforementioned reason, this present study aimed at assessing the global research interest and productivity in MAP via bibliometric analytic frameworks from authors, country, institution, international networking, conceptual frameworks and theme developmental perspectives. Also, it undertakes growth analysis of MAP in the different areas such as scientific subject areas, institution participations, research topics and autoimmune diseases. These quantitative and qualitative approaches aimed at creating awareness and arouse interest in MAP research and serve as primer of information database for researchers on where to collaborate and seek appropriate resources for their research.

## 2. Materials and Methods

### 2.1. Mycobacterium avium Subsp. Paratuberculosis (MAP) Data Sources

**Figure 1:**
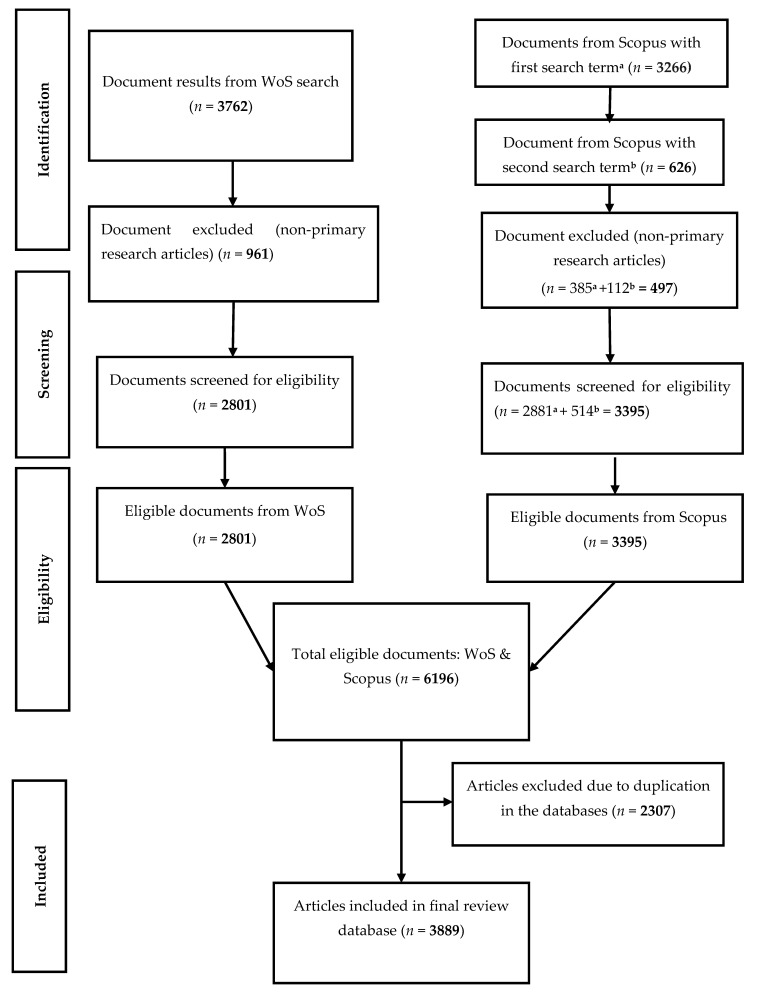
Preferred Reporting Items for Systematic Reviews and Meta-Analyses (PRISMA) flow chart for mining and screening *Mycobacterium avium* subsp. *paratuberculosis* (MAP) documents from 1911 to 2019 [22]. a = Scopus search with ‘paratuberculosis*’; b = Scopus search with ‘Johne’s disease’.

The study retrieved MAP-related peer-reviewed primary articles from the Scopus database and the Web of Science (WoS) core collections from 1911 to 2019 according to the method prescribed by the Preferred Reporting Items for Systematic Reviews and Meta-Analyses (PRISMA) [22]. The two databases were searched separately and the datasets combined with duplicated articles marched as one. The search was performed as title-specific algorithm on 29 February 2020, 17:48:39 (GMT +2). Primarily, the search terms ‘*paratuberculosis*’ or ‘Johne’s disease’ were used solely in mining the MAP metadata. For the Scopus search, the algorithms were “TITLE (‘*paratuberculosis*’) AND PUBYEAR < 2020” and “TITLE (’Johne’s disease’) AND PUBYEAR < 2020”. The WoS search algorithm was: “TITLE: (‘*paratuberculosis*’ or ‘Johne’s disease’), Timespan: 1990–2019. Indexes: SCI-EXPANDED, SSCI, A&HCI, CPCI-S, CPCI-SSH, BKCI-S, BKCI-SSH, ESCI, CCR-EXPANDED, IC.”. Primary research articles only were included in the analysis Other documents were excluded for the reasons that they had been pre-article items and might be published in other forms (duplicated publishing) or post-publication synthesis of primary articles (Figure 1). Manual validation of the 15 top articles was further done to ascertain the specificity and efficiency of the search algorithm in recovering the MAP metadata from the two databases. The articles were downloaded as tab-delimited (Win, UTF-8), BibTeX (bib), and comma separated (CSV Excel) file formats for data pre-analytics section.

### 2.2. Pre-Analytic and Bibliometric Assessment of the Data

A data pre-analytic protocol was performed on the hybrid dataset from the two databases. This included extraction and normalization of all bibliometric attributes in the dataset as described previously [23]. The attributes of interest involved authors’ names, affiliations/institutions, country, article source, and keywords. Pre-analytics focused on minimising errors and inflation in outputs by watching for duplication, variations in spelling, and synonymic forms etc. Specifically, authors’ names and keywords were checked for spelling inconsistencies, synonymic forms, UK/USA variant spelling, multi-occurrences of one keyword in an article, etc.

The standardised data were analysed for performance indicators including trend, descriptive indices/rates related to article (annual production, average citations/articles), author rates (number of authors, author appearances, authors of single-authored articles, authors of multi-authored articles, single-authored articles, articles/author, authors/article, co-authors/articles), and productivity (20 top productive authors, the 20 top institutions, 50 top countries, total citations per country and 20 top publishing journals). In addition, quality indicators’ estimates such as articles, authors, sources and countries significance in the MAP research domain, collaboration index, number of citations, H-index, and impact factors were considered.

Factorial mapping of the most cited documents or articles with the highest contributions in MAP research was performed through co-word analysis of author-keywords via multidimensional scaling (MDS) [23].

### 2.3. Determination of Growth of MAP Research

A growth analysis of MAP research in relation to autoimmune diseases (ADs) and some hot research topics in veterinary, food and environmental microbiology was carried out. The ADs considered include type 1 diabetes mellitus (T1DM)/diabetes, multiple sclerosis; sarcoidosis, thyroiditis (Hashimoto’s thyroiditis, transthyretin), psoriasis, ulcerative colitis, Parkinson’s disease, arthritis (Blau syndrome; rheumatoid arthritis), and lupus. Also, the hot topics evaluated ranged from environment (river, freshwater, stream, pond), virulence (and resistance), osteopontin, livestock (goat, sheep, cattle, cow, bovine, camel), wild animals (wild, rat, cervus, deer, fox, boar, weasel), soil/pastures, children (paediatric, infant, neonate, child), milk and milk products, animal wastes (faeces, urines and manure), wastewater (sludge, slurry, digestate), microbiota and feed.

The topics were identified via their average growth rate (AGR) based on authors’ keywords from 2017–2019. The topic’s AGR was assessed as described by Ruiz-Rosero et al. [24] according to Equation (1).

(1) $$\mathrm{AGR}=\left(\overset{2019_{e}}{\underset{i=2017_{s}}{\sum }}D_{i}-D_{i-1}\right)/\left(2019_{e}-2017_{s}\right)+1$$

where: AGR = average growth rate; 2017_s_ = start year; 2019_e_ = end year; D_i_ = number of documents in 2017.

### 2.4. Determination of Scientific-Networking in MAP Research

Resource- and intellectual-sharing forms the basis for scientific-networking and collaborations. This study determined different collaboration networks in *MAP* research. Collaboration networks could either be between authors, institutions or countries as bipartite vectorial matrices of a typical $Netw=M\times M^{T}$ where *Netw* (a symmetrical matrix *M* = *M^T^*) is composed of: 1. Articles × Authors (authors’ collaboration network (ACN)), 2. Articles × Countries (country collaboration network (CCN)), and 3. Articles × Institutions (institution collaboration network (ICN)), and *M* is a bipartite network matrix. Characteristically, the collaboration network has its edges/nodes constituting authors/institutions/countries and the linking lines the corresponding basis/means of relationships (knowledge-sharing/resource-sharing) between them. The networks were graphed using the Fruchterman force-directed algorithms with Jaccard’s similarity index normalization [25]. The performance of each network was assessed via classical network statistics such a size (NZ), density (NDE), diameter (NDI), degree centralization (NDC), transitivity (NT) and average path length (NAPL), closeness centralization (CC), betweenness centralization (BC), eigenvector centralization (EC) and network degree distance (NDD).

### 2.5. Software

Data analysis was undertaken in R and python programme environments. All analysis was implemented based on bibliometrix package [26] in Rstudio v1.2.5042 with R v.3.6.2 (RStudio Team, 2018; Boston, MA, http://www.rstudio.com/), ScientoPy v.2.0.3 package (Ruiz-Rosero et al. 2019) and Excel 2016.

## 3. Results and Discussion

This section presented MAP research bibliometric output from 1911–2019 and discussed systematically its growth from 2017–2019.

### 3.1. MAP Bibliometrics

#### 3.1.1. Characteristics of MAP Research Documents

The features of MAP publications from 1911–2019 is presented in Table 1. A total of 6662 authors participated in MAP research in the period with a total of 18,666 author appearances. The studies were published in 581 journal outlets. Document-associated rates distributed as 20.65 average citations/documents, 348 single-authored documents, 0.584 documents/author, 1.71 authors/document, 4.8 co-authors/documents and 1.82 universal collaboration index. A careful evaluation of the author-document rates and the collaboration index (CI) suggests a poor performance of MAP research over the period. A booming research field generally have higher values of author-document rates and CI.

#### 3.1.2. Language Diversity in MAP Research Communications

**Figure 2:**
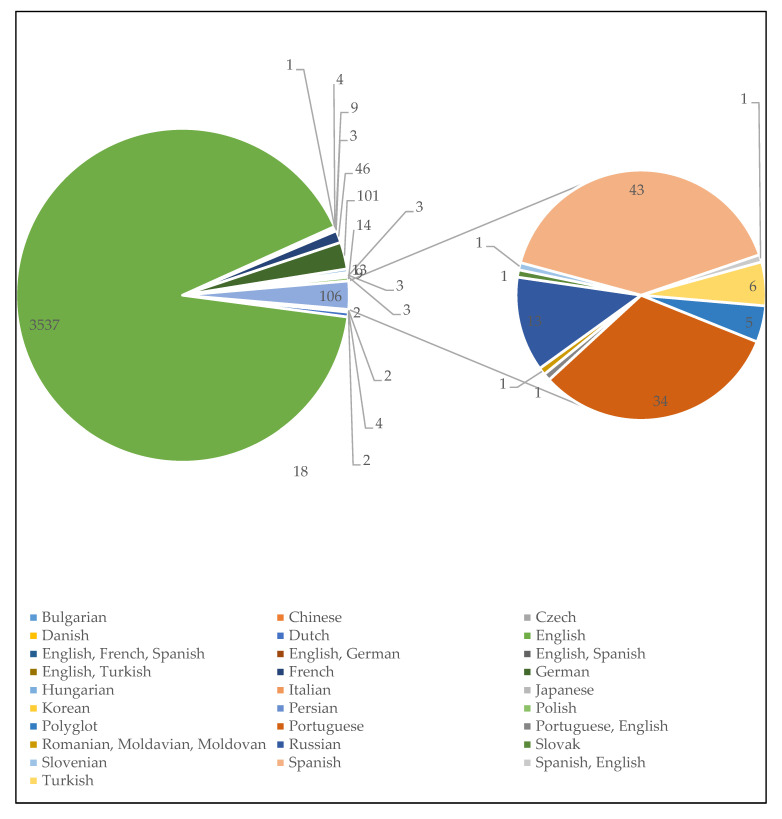
Publication language distribution of *MAP* output research from 1911 to 2019. Language Frequency % of 3889. Bulgarian (0.05%), Chinese (0.05%), Czech (0.10%), Danish (0.05%), Dutch (0.46%), English (90.95%), English, French, Spanish (0.03%), English, German (0.10%), English, Spanish (0.23%), English, Turkish (0.08%), French (1.18%), German (2.60%), Hungarian (0.33%), Italian (0.23%), Japanese (0.08%), Korean (0.08%), Persian (0.08%), Polish (0.36%), Polyglot (0.13%), Portuguese (0.87%), Portuguese, English (0.03%), Romanian, Moldavian, Moldovan (0.03%), Russian (0.33%), Slovak (0.03%), Slovenian (0.03%), Spanish (1.11%), Spanish, English (0.03%), and Turkish (0.15%).

Figure 2 depicts the distribution of MAP documents according to language of research communication or publication. Most documents were published in English (*n* = 3537, 90.95%) while other languages include Bulgarian (*n* = 2, 0.05%), Chinese (*n* = 2, 0.05%), Czech (*n* = 4, 0.10%), Danish (*n* = 2, 0.05%), Dutch (*n* = 18, 0.46%), English, French and Spanish (*n* = 1, 0.03%), English and German (*n* = 4, 0.10%), English and Spanish (*n* = 9, 0.23%), English and Turkish (*n* = 3, 0.08%), French (*n* = 46, 1.18%), German (*n* = 101, 2.60%), Hungarian (*n* = 13, 0.33%), Italian (*n* = 9, 0.23%), Japanese (*n* = 3, 0.08%), Korean (*n* = 3, 0.08%), Persian (*n* = 3, 0.08%), Polish (*n* = 14, 0.36%), Polyglot (*n* = 5, 0.13%), Portuguese (*n* = 34, 0.87%), Portuguese and English (*n* = 1, 0.03%), Romanian, Moldavian and Moldovan (*n* = 1, 0.03%), Russian (*n* = 13, 0.33%), Slovak (*n* = 1, 0.03%), Slovenian (*n* = 1, 0.03%), and Spanish (*n* = 43, 1.11%), Spanish and English (*n* = 1, 0.03%), and Turkish (*n* = 6, 0.15%). The dominance of English language could be understood from the point of view that most publishers only publish articles written in English language and that the English language is widely accepted among the scientific community. The appearance of articles published in other language promote(s) awareness of the pathogen among non-English elites. Bilingual and multilingual publications also improved coverage and effective communication of MAP scientific results. It is important that the language of publication embraces regional languages for proper dissemination of research findings between ‘gowns’ and ‘towns’ in cases of emerging and infectious diseases to aid various campaigns and efforts to halt their further spread.

#### 3.1.3. Annual Productivity of MAP Research Landscape

**Figure 3:**
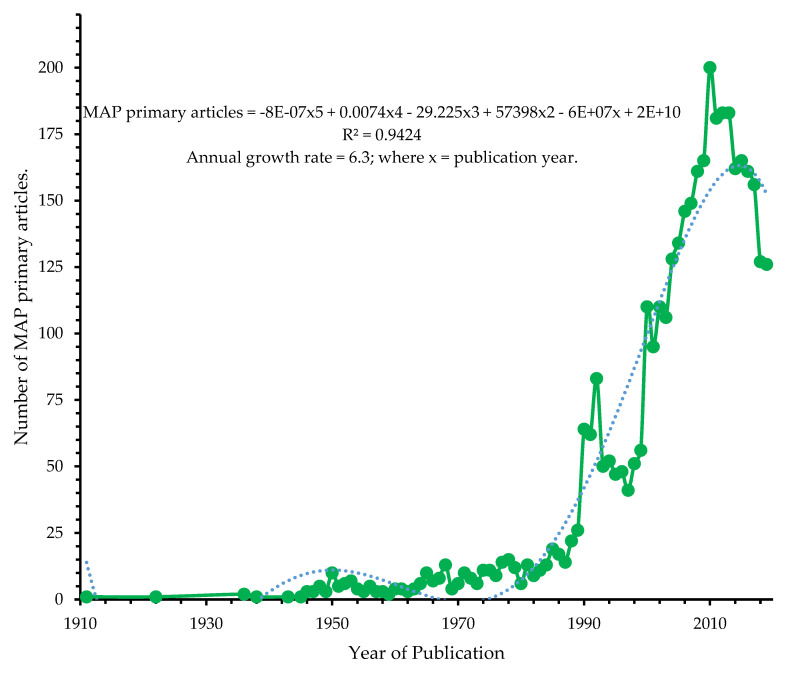
Annual scientific output related to *M. avium* subsp. *Para tuberculosis* research from 1911–2019.

The general trends of MAP research from 1911–2019 had 6.3% annual scientific growth rate. The annual growth can be predicted by a 3rd order polynomial relationship between total number of papers and the year (Figure 3). The observed annual growth rate of MAP research revealed low attention its gained annually at global level compared with localized growth in some regions such as Europe and Asia. More attention is required to create awareness and arouse research interest, especially in low-resource nations, where MAP research is currently low due to technical know-how, the inherent low growth attribute of MAP, and instrumental challenges.

#### 3.1.4. Authors’ Productivity

The top 20 productive/active researchers in MAP research from 1911– 2019 are presented in Table 2. The first 3 authors (Articles; H-index) were Whittington, R.J. (*n* = 143, 34), Collins, M.T. (*n* = 130, 35) and Stabel, J.R. (*n* = 125, 30) who occupied 1st, 2nd, and 3rd positions respectively. Other researchers whose works had a positive growth rate (30%) in MAP research in the last 3 years (2017–2019) included Bannantine, J.P. (109 articles), Garrido, J.M. (50 articles) and Wells, S.J. (49 articles). The influence wielded by these authors stemmed from the studies that introduced molecular diagnostic biomarkers/method such as IS900-based polymerase chain reaction, taxonomy, and pathogenesis markers in MAP research [27], that revealed environmental persistence of MAP up to 55 weeks in a dry shaded environment, and 9 to 24 weeks on grass [17], hidden subclinical symptoms and long-term i.e., 2–5 years’ latent period of MAP infections in cattle [28], antibody-based diagnosis or detection of MAP in bovine *paratuberculosis* [29], and IS1311 sequence typing of MAP from different sources [30] among others.

#### 3.1.5. Most Cited/Articles with Highest Contributions in MAP Research

The top cited titles (based on TC; TC/Year) in MAP research from 1911–2019 with their total citations, and total citations per year, are shown in Table 3. The papers present fundamental evidences, challenges and research breakthroughs related to the pathogen. First, economic losses due to MAP infection in the US dairy industry (439 citations) [6], culture of MAP from blood of Crohn’s disease patients (434 citations) [31], characteristic sequence of IS900 in MAP and MAP isolates from human Crohn’s disease (370 citations) [32], MAP DNA in tissue from Crohn’s disease condition (322 citations) [33] as zoonotic implication in human diseases, MAP complete genome sequence (316 citations) [34], MAP cultured from supramammary lymph nodes, milk and subclinical MAP infection in cows (315 citations) [35], ileocolonic mucosal biopsy specimens detection and verification of MAP in clinical Crohn’s disease and healthy individuals (307 citations) [29].

#### 3.1.6. Country’s Productivity in MAP Research

Table 4 shows the performance of 70 countries in MAP research from 1911–2019. The USA and Australia occupied the first and second positions in terms of total number of articles, and frequency of publication (FP). While the USA had 887 (22.81%) articles, and 26.72% FP, Australia pooled 236 (6.07%) articles, and 6.07% FP. The observed retention of positions by the USA and Australia in terms of the two measures informed the dedication of the countries to MAP studies, quality and influence that their research output have in MAP research landscape. Single-country articles were prevalent. The SCA/articles (%) valued 100% in countries such as Pakistan, Slovenia, South Africa, Venezuela, Bulgaria, Peru, Philippines, Albania, Algeria, Costa Rica, Ghana, Indonesia, Iraq, Kuwait, Nepal, Russia, Singapore and Thailand. This further confirms that MAP research lacks international collaboration among most nations, especially, low-resource countries.

Based on total citations, the USA (26418 citations), United Kingdom (7723 citations) and Australia (6014 citations) ranked 1st, 2nd and 3rd respectively. But considering average articles’ citations (AACs), the United Kingdom (40.22 AACs), Netherlands (30.41 AACs), USA (29.78 AACs), Denmark (28.59 AACs) and Ireland (28.02 AACs) occupied the first 5 positions (Table S1). Summarily, MAP research is limited to North America (USA, Mexico, Canada), Europe (Germany, United Kingdom, Czech Republic, Ireland, Spain, Denmark, Norway, Portugal, Belgium, Sweden, Poland, Slovakia, Italy, Iceland, Finland, Austria, Switzerland, Netherlands, France, Greece, Hungary, Slovenia), Asia (India, Korea, Japan, China, Pakistan, Taiwan, Singapore, Iran, Israel, Jordan), South America (Argentina, Chile, Brazil, Peru, Venezuela, Colombia), Oceania (Australia, New Zealand), Turkey(Europe, Asia) and merger contributions from Africa (Egypt, South Africa, Sudan, Uganda, Morocco, Algeria, Cameroon and Ghana). The economic impacts of MAP and associated infections and ADs in low-resources countries need assessment to understand agro-economic losses and public health risk due to the pathogen. The lack of MAP research in most low-resource countries is directly linked to a lack of available facilities and technical know-how for MAP research. MAP research requires sophisticated instrumentations because of its inherent slow growth and difficult-to-cultivate characteristics. Many research questions are needed to answer problems related to MAP in human ADs, herds and herders in the resource-limiting countries given the wealth of pastoral activities in the nations.

#### 3.1.7. Journal’s Productivity in MAP Research

The top 20 important journal outlets on MAP research from 1911–2019 is shown in Table 5. Twelve out of the 20 journals have their scopes in veterinary research. These include *Veterinary Microbiology, Preventive Veterinary Medicine, Veterinary Immunology* and *Immunopathology, American Journal of Veterinary Research, Journal of Veterinary Diagnostic Investigation, Australian Veterinary Journal, Journal of the American Veterinary Medical Association, Veterinary Record, Veterinary Research, Small Ruminant Research, Pesquisa Veterinaria Brasileira* and *Acta Veterinaria Scandinavica*.

Other journals such as *Journal of Dairy Science, Journal of Clinical Microbiology, Applied and Environmental Microbiology, Infection and Immunity, Journal of Comparative Pathology, Clinical and Vaccine Immunology* and *Berliner Und Munchener Tierarztliche Wochenschrift* deal in food, human/animal related infectious and environmental health, and multidisciplinary research (Plos One). The impact factor (IF) of the journals valued above was from 1.0 to 4.959 excluding the *Australian Veterinary Journal* (0.887 IF) and *Veterinarni Medicina* (0.636 IF). Also, the H_index of the journals according to the local collections varied from 8 to 49. Elsevier BV/Ltd. (Netherlands/UK) was the most active publishing housing in MAP research with 7 journals. The various journal outlets underscore the public health importance of MAP infections from the one-health perspective. The associated impact factors also attest to the quality of the journals.

### 3.2. Growth of MAP Research from 2017–2019

#### 3.2.1. Growth of MAP Research Related to Discipline Classification

Table S2 presents growth of MAP research in relation to 52 disciplines. Veterinary science and microbiology occupy the 1st and 2nd positions, with 1450 articles (67 h-index) and 702 articles (72 h-index) respectively. This reveals the veterinary and microbiological public health importance of MAP. There was positive growth of MAP research only in the following subjects with h-index ranging from 18 to 52: food science and technology (*n* = 234, 70%), immunology (*n* = 373, 30%), agriculture (*n* = 336, 30%), pathology (*n* = 52, 30%) and research and experimental medicine (*n* = 41, 30%). This is directly related to various research efforts in elucidating MAP’s disease pathophysiological and immunological biomarkers, and agricultural preventions/amelioration of MAP economic losses on farms.

Also, a 0% growth rate of contribution was observed in the last 3 years in MAP research from Instruments and Instrumentation, Entomology, Business and Economics, Mathematics, Water Resources, Biodiversity and Conservation, Electrochemistry, Evolutionary Biology, Fisheries, Nutrition and Dietetics, Oncology, Paediatric, Rheumatology and Spectroscopy among other disciplines. More research from the disciplines are required as their previous activities highlighted germane contributions and concerns that might be helpful in the war against MAP infections. For instance, entomological studies implicated flies as significant vehicles in the dissemination of MAP infections [38,39,40]. Insects such as *Blatta orientalis* (Oriental cockroach), *Calliphora vicina*, *Lucilia sericata* (blowflies) and *Eristalis tenax* (syrphid flies) have been implicated in the spread of MAP [38,39,40].

Furthermore, the priority for the development of instruments and instrumentations for point-of-care diagnosis of MAP infection [41,42,43] still needs emphasis in research. Present diagnostics approaches have a long-term turnaround time coupled with costly, advance equipment and skilful trainings, which are not easily come by in resource-limiting countries. The available point-of-care diagnostics proposed and experimented for MAP in literature include conductometric biosensor aimed at detecting MAP IgG in 2 min. [41], conductometric biosensors coupled with immunomigration technology for detection of the MAP IgG antibody [42], and an electrochemical impedance spectroscopy biosensor for MAP’s DNA detection [43].

The occurrence of MAP has been reported in water resources including water treatment works and their effluents and kitchen faucet biofilm samples up to incident rates of 26%–58% [44,45]. MAP has been detected in colorectal cancer, and might contribute to its development [46]. More studies are, therefore, needed to understand the role of MAP from an oncology perspective.

#### 3.2.2. Growth of MAP Research per Country

Percentage average growth of MAP research in different countries in the last 3 years is presented in Table S3. Highest growth rate was found in Germany and Egypt, each with 170% AGR in 2017–2019. In countries such as the USA, Spain, Chile, Switzerland, Poland, Saudi Arabia, Thailand, Uruguay, Algeria and Bulgaria, the AGR of MAP research ranged from 30% to 100%. The MAP AGR from these countries represent 8% to 100% documents published in the last few years (2017–2019). Other nations have no growth or declined AGR in MAP research in the last 3 years. The countries with positive and high AGR show the countries’ continuous efforts in MAP research. Such countries could be a destination target for students and researchers that have interest in MAP. Also, they could offer collaboration opportunities for researchers from other nations. Generally, every country should increase their research towards combating MAP infection in human autoimmune diseases, herds and herders who are prone to infections.

#### 3.2.3. Growth of MAP Research Based on Institution Participations

Table S4 presents the growth of MAP research based on institution participations in the last 3 years. The following institutions with 30% or 70% AGR could serve as target destination for individuals with interest in MAP research due to active works from the institutions: Univ. Sydney Australia; Univ. Minnesota, United States State Univ., United States; Michigan State Univ., United States; Univ. Coll. Dublin, Ireland; Univ. Calif Davis, United States; Shiraz Univ., Iran; AgResearch, New Zealand; Wageningen Univ., Netherlands; Univ. Florida, United States; TEAGASC (The Agriculture and Food Development Authority), Ireland; Anim Hlth Ireland, Ireland; Ist Zooprofilatt Sperimentale Lombardia and Emilia, Italy; Univ. Antioquia, Colombia and Univ. Complutense Madrid, Spain.

### 3.3. Trend and Growth of MAP Related to Some Hot Research Topics

MAP research attracted positive growth from 2017–2019 in topics related to wildlife (30%), environments (100%), virulence (70%), disease resistance (100%), meat and meat products (30%), wastewater/waste milk (30%) and digester slurry/sludge (30%) (Table 6). However, negative growth was observed in the following topics: livestock (−30%), milk and milk products (−130%), faeces (−70%), water (−30%), and microbiota (−30%). Also, no growth in MAP research related to manure, pasture/pasteurisation, sewage and urine in the last 3 years.

#### 3.3.1. Growth of MAP Research in Wildlife-Related Discipline

The observed growth of MAP research in wildlife-related topics further support the existence of MAP infections in wild herds. Also, the possibility of zoonotic transmission of MAP from wild herds to domestic/zoological reserves’ herds and from either herd forms to humans could not be overemphasised [47] most especially, in countries (e.g., South Africa) where domestic herds and wild games are farmed together on the same piece of land. Some recent studies related to MAP infections in wild games include deer in Bulgaria and Chile [48,49], 34.4% incidence of MAP in *Anas* species (wild duck) populations in Korea [50], and wild-born *Procavia capensis* (rock hyraxes) imported from South Africa to Germany [47].

#### 3.3.2. Growth of MAP Research Related to Environmental Milieu

The increase in the number of MAP studies related to environments could stem from its significant persistence across environmental continuums and matrices such as these have generated public health concerns about the spread of MAP. Also, environmental samples offer a cheap and convenient method for assessing MAP-infected herds [51]. For instance, MAP was detected on spatiotemporal scales in the River Tywi catchment with persistence over a decade with little change [52]. In the study, MAP concentration in the river equals to 10^−8^ cell equivalents/L consistently over some finer timescales and in-stream influence of small wastewater treatment plants had insignificant effect on its levels which eliminated probable ingress of human-associated MAP. Also, the aerosol spread of MAP was established in natural river foams in the study. Environmental sampling and assessment protocols for MAP detection has been developed [53,54]). Other recent, MAP studies related to environments include environmental detection of MAP in some matrices such as drylot pens, dairy herds’ paddock, milking facilities, anaerobic digestion of slurry from MAP-infected farm, manure storage, lactating cow surroundings, housing (freestall, loose housing, or tiestall), liquid manure and boot swabs [18,51,52,53,54,55,56,57,58,59].

#### 3.3.3. Growth of MAP Research Related to Virulence and Disease Resistance

MAP virulence and its animal disease resistance enjoyed positive research growth in the last 3 years. Research into MAP virulence is rooted in the desire to understand pathophysiological effects of MAP in different life forms and to establish clinical and subclinical biomarkers of MAP infections and diseases. These researches seek to determine molecular resistance, susceptibility gene markers or factors that drive the establishment of MAP infections in hosts [60,61,62,63,64,65,66,67]. Growth related to MAP disease resistance research underscored the inclination towards selection of animal breeding stocks that are capable of high productivity in the event of MAP infections on-farm to minimize husbandry failures and economic losses. In animals, this trait is desired as a mean for selection of animal breeds to reduce economic loss due to MAP infections especially, in resource-limiting countries, where underlying complexities of MAP, slow growth rates, and lack of diagnostic technique/technical know-how hinders MAP research and controls.

Recent studies tailored towards elucidation of MAP virulence biomarkers include exploration of mathematical modelling such as general linear and neurofuzzy logic to inform how host variables (e.g., macrophage, sheep, cattle etc.), MAP genotype and post-infection time contribute to MAP infection virulence in an in vitro model [68]. In the study, Alonso-Hearn et al. [68] revealed different susceptibilities and distinct virulence profiles of bovine and ovine macrophages to MAP based on intracellular-macrophages growth or persistence capability, and found that non-domesticated animal and bovine MAP isolates are more virulent than the domestic animal MAP isolates. Bo et al. [69] and Slavin et al. [70] identified protein kinase G (PknG) and protein tyrosine phosphatase A (PtpA) as possible virulence biomarkers of MAP’s involvement in rheumatoid arthritis pathogenesis, and multiple sclerosis and neuromyelitis optica spectrum disorder respectively. Other studies that sought to understand the molecular/genetic basis of MAP infection susceptibility or resistance in the last 3 years include Kumar et al. [65], Kumar et al. [66] and McGovern et al. [67].

Genetic polymorphisms or single nucleotide polymorphisms in bovine *CLEC7A*, toll-like receptor (TLR2/TLR4) genes and putative susceptibility quantitative trait loci (*ZBTB20, KALRN, LPP, SLA2, DNAJC6, ZDHHC14, FI3A1, LRCH3, HAS2*, and *SNX1* genes) are relevant genetic biomarkers of MAP susceptibility or resistance in bovines [65,66,67]. Single nucleotide polymorphisms (SNPs) rs110353594 and s41654445 in the bovine *CLEC7A* gene and SNPs rs8193046 and rs8193060 in toll-like receptors have been identified as genetic markers of greater resistance to MAP infections [65,66].

Transcriptome studies of monocyte-derived macrophage cultures from uninfected red deer previously confirmed resistant or susceptible to MAP infection revealed a more discontinuous gene expression pattern in macrophages derived from susceptible phenotype compared with macrophage cells from resistant phenotypes in relations to the number of genes up(down)regulated. Upregulation of chemotaxis genes (*CXCL10, CSF3,* and *CCL8*) and type I interferon signalling genes (*IFIT1, IFIT2, RSAD2, ISG12, ISG15, HERC6*, and *USP18*) were greater in susceptible animals’ macrophages compared to resistant animals’ derived macrophage cells in reaction to in vitro MAP infection [61]. Thus, serve as biomarkers for disease susceptibility or resistance. Cattle have been grouped into 3 classes based on their disease resistance or susceptibility profiles, namely non-MAP shedders (herd negative by faecal culture), low-MAP shedders (herd that shed MAP sporadically small ( ≤19 colony-forming units (CFU)) and remained clinically healthy) and high-MAP shedders (herd that repeatedly shed MAP ≥10 CFU with gradual deterioration of health conditions).

#### 3.3.4. Growth of MAP Related to Anaerobic Processes

The role of anaerobic digestion on MAP reduction in manure emerged in the last two years [18,55]). The positive growth of MAP research in anaerobic digestion of animal manure and slurry ensured MAP reduction and improved the quality of digestates as biofertilizers for pasture and agricultural soils compared with untreated ones [18,55]). A-two-stage anaerobic digestion system for biofertilizer production has been identified to improve final digestate qualities and reduce the risk of MAP contamination of the environment and agricultural produce compared to a single-stage digester [55].

#### 3.3.5. Growth of MAP Research Related to Waste Milk Feeding in Animals

MAP research coupled with waste milk feeding in animals had 30% growth between 2017–2019. This is due to the possibility of MAP transmission due to feeding of waste milk to calves [71,72]. Leao et al. [71] found that 89.2% of farms enrolled in their study fed waste milk to calves, without pasteurisation except one farm. The study found 51.5% of the farms had a positive MAP result in the waste milk fed to calves. Other factors involved in MAP positivity from feeding waste milk to calves include animal origins (the incidence of MAP in farm with herds from a single origin was 3.5 times higher and 1.9 times higher in farms with herds from multiple origins), and calving area (multiple cow calving increase the risk of MAP positivity by 1.5 compared with single pens) [71]. Generally, waste milk pasteurisation has been found inadequate to limit MAP transmissions to calves as viable cells (≥1000 MAP cells/mL) were still present after a short pasteurisation period [72].

#### 3.3.6. MAP and Other Research Topics

The study found negative growth in MAP studies related to livestock, milk and milk products, faeces, water, and microbiota and no growth/research related to sewage, pasture and urine in the last 3 years. Studies of MAP related to water and water systems in the period involved detection of MAP in environmental amoebae (*Rosculus* species) from sampled water from MAP-infected farms [20] and optimized recovery and decontamination of MAP from spiked water sediments [44]. MAP research related to water and water distribution is needed as this medium serve as possible transmission route of high public health threats to humans and livestock. More research is required to monitor the possible and continuous roles of milk and milk products, microbiota, sewage, pasture and urine in the transmission and pathogenesis of MAP.

### 3.4. Growth and Trend of MAP Research Related to Autoimmune Diseases

This section presented overview of MAP research output from 1911–2019 related to ADs and discussed systematically its growth from 2017–2019 (Table 7).

#### 3.4.1. Growth of MAP Research Related to Type 1 Diabetes Mellitus (T1DM)

MAP has been report detected in 11–22 million type 1 diabetes mellitus (T1DM) patients worldwide [73]. Growth analysis however, revealed the AGR of MAP research related to T1DM in the last 3 years to be 70% and 10 documents (Table 7). Various biomarkers of MAP infection in T1DM include MAP3738c and MptD [74,75], cross reaction between MAP Heat shock protein 65 K (Hsp65) and human T1DM patient sera (glutamic acid decarboxylase 65 K), serum Th1 (CXCL10) chemokine in infants [76], and selenium-dependent glutathione peroxidase activity [77]. No link has been observed between transthyretin (TTR) plasma levels and T1DM MAP exposed patients; rendering TTR to be not a good biomarker candidate for T1DM [78]. Bo et al. [79] has also reported the correlation between humoral reactivity to MAP and lipoprotein levels in T1DM and found that HDL (high density lipoprotein), LDL (low density lipoprotein)/VLDL (very low density lipoprotein) and total cholesterol (TC) levels were significant differences between patients and healthy controls (*p* < 0.0001). MAP-positive patients had lower HDL levels compared with MAP-negative subjects and opposite trends in the case of LDL/VLDL concentrations (*p* < 0.05) [79].

#### 3.4.2. Growth of MAP Research Related to Multiple Sclerosis Diseases

MAP was significantly detected in higher numbers in 2.5 million multiple sclerosis patients [73]. However, MAP research coupled with multiple sclerosis had 0% growth from 2017–2019 and only 10 documents from 1911 till 2019 (Table 7). This suggests slow or grossly inadequate publication reports of MAP research in affinity with multiple sclerosis conditions in the last 3 years. Meanwhile, MAP has been shown to trigger multiple sclerosis (MS) by various investigations [75,76,79,80,81,82,83]. For instance, a high prevalence of the MAP_0106c protein (MAP121_132), Epstein–Barr virus (EBV) nuclear antigen1(400–413), and myelin basic protein (85–98) has been found among MS patients in comparison with healthy individuals. Also, the study shows that Epstein–Barr virus nuclear antigen1, and myelin basic protein antibodies cross-react with MBP85–98 via molecular mimicry [80]. Antibodies recognising EBNA1(400–413) and MAP (121–132) cross-react with MBP85–98, possibly through a molecular mimicry mechanism. Antibodies of self-epitopes in T1DM cross-recognise MAP and EBV peptides lending credence to the possible role of the pathogens in the autoimmune condition. The prevalence of MAP and EBV DNA among 119 Sardinian MS patients was shown as 27.5% and 17.3%, respectively; with extremely high body fluid immunologic response against MAP recombinant protein, MAP FprB, known as human myelin P0 homologous [81]. However, the studies on MAP in the MS condition have been limited to very narrow geographic location such as Sardinia. Investigation also detected MAP2694 antibodies and MAP DNA in 28.2% (123/436) and 15.6% (68/436) MS patients based on an enzyme-linked immunosorbent assay (ELISA) and IS900-specific polymerase chain reaction (PCR) in Sardinia [84]. Also, MAP_2694_295–303_ serological marker had 30% incidence rate among Japanese MS patients [84]. Highly levels of antibodies against tyrosine phosphatase A and protein kinase G MAP virulence factors were found in MS patients and neuromyelitis optica spectrum disorder (NOSD) suggesting that these patients have been exposed/infected with MAP and the possible role of MAP in NOSD condition [70].

#### 3.4.3. Growth of MAP Research Related to Sarcoidosis

In 1.9 million sarcoidosis patients, MAP was significantly reported in higher numbers [73]. However, the MAP research/published documents in the last 3 years only witness 0% AGR and 2 cumulative documents throughout the entire study period (Table 7). However, the role of MAP in sarcoidosis has been controversial; while Lisby et al. [85] found no role of MAP in sarcoidosis, Celler [86] reported a complete metabolic resolution of cardiac sarcoidosis following a year of MAP antibiotics treatment course, thus, providing a clue to a possible link between sarcoidosis and MAP infection.

#### 3.4.4. MAP Importance in Thyroid Disorders (TDs)

The aetiological affinity and role of MAP in Hashimoto’s Thyroiditis patients is still a subject of debate [73]. But, growth analysis of MAP research connected to TDs had only 2 documents with 0% growth rate in the last 3 years (Table 7). This shows that MAP research linked to TDs has not gained ample research attention. However, Gupta et al. [87] observed 36.8% (*n* = 28/76) MAP sero-positive incidence in TD patients based on IS900 gene PCR.

#### 3.4.5. Growth of MAP Research Related to Psoriasis

The possible role of MAP in psoriasis has been postulated and had attracted calls for research as at 4 August 2017 by Humanpara Foundation [73]. However, this has not been translated into research efforts. No research document focused on MAP coupled with psoriasis in the last 3 years (2017–2019) based on the current dataset (Table 7). Experimental/clinical demonstrations are needed to provide evidence-based roles of MAP infection in psoriasis and device solutions to such occurrence.

#### 3.4.6. Growth of MAP Research Related to Irritable Bowel Syndrome (Crohn’s Disease and Ulcerative Colitis)

Detection of MAP in 2–4 million patients with ulcerative colitis (UC) has been documented/speculated upon [73]. However, only 13 research documents considered MAP in connection to UC throughout the study period [88,89,90,91,92,93,94,95]. Growth of MAP research linked with UC had a negative growth rate of –30% (Table 7). This reveals a declined state of research efforts related to the role of MAP in UC and possible treatment courses. An elevated secretion of TNF (tumour necrosis factor)-alpha in Crohn’s disease correlates with molecular biomarkers of MAP infections [96]. Also, the presence of MAP correlates significantly with higher levels of tumour necrosis factor-a in MAP-positive Crohn’s disease patients, higher than MAP-positive UC and MAP-positive irritable bowel syndrome compared with MAP-negative and healthy individuals [96]. Findings from Ren et al. [91] attributed altered T-cell function with MAP infection in Crohn’s disease. Furthermore, Crohn’s disease patients’ susceptibility to MAP has been connected to SLC11A1 [94]. However, some works did not support MAP’s aetiological role in Crohn’s disease [97,98]. Thus called for further re-examination or validation of MAP’s definitive causal role in Crohn’s diseases. The mere high loads of MAP in human intestinal biopsies of Crohn’s diseases has been refuted as evidential confirmation of MAP aetiological role in Cohn’s disease [95].

#### 3.4.7. Growth of MAP Research Related to Parkinson’s Disease

The role of MAP has been implicated in Parkinson’s disease (PD) [73]. However, more studies are needed to establish the actual causative roles of MAP in PD. From growth analysis, only 1 paper reported a study that linked MAP with PD (Table 7). A negative −30% growth rate of MAP research linked with PD depicts declined research activities into the causal affinity between MAP and PD. Arru et al. [99] reported a detection of MAP (PCR) and MAP antibodies (MAP3865c by ELISA) in PD patients. However, the study could not demonstrate cross reaction between MAP antibodies and human homologous Znt proteins (ZnT3 and ZnT10) known to play major role in PD pathogenesis. Future pursuit of research elucidation of the early hypothesis of ineffective xenophagy or autophagy exhaustion as a permissive environment for MAP infection and resultant PD pathology might provide useful insights to the causal relationship between MAP and PD [100].

#### 3.4.8. Role of MAP Arthritis and Osteoporosis (Blau Syndrome; Granulomatoud, Rheumatoid Arthritis)

The year 2017–2019 witnessed 100% growth rate of studies that implicated MAP in the aetiology of rheumatoid arthritis (Table 7). The total of 4 documents identified were published in the last 3 years and implies MAP research related to this subject started gaining attention from 2017. MAP infection and osteoporosis in rheumatoid arthritis correlated with TNF Receptor Superfamily 1B (TNFRSF1B:rs3397) polymorphisms in particular, and its downregulation by rs3397 worsens inflammation and leads to osteocalcin deficiency [101]. The interferon regulatory factor 5, single nucleotide polymorphisms in T-cell negative-regulators, protein tyrosine phosphatase A and protein kinase G are biomarkers with significant correlation between rheumatoid arthritis and MAP infections [69,79,101,102].

#### 3.4.9. Contributions of Osteopontin in MAP

Five documents reported the role of osteopontin in MAP infections in the study period. Only 40% of the papers were published in the 2017–2019 (Table 7). MAP research relate to osteopontin had 70% growth rate in the last 3 years The role of osteopontin in the regulation of MAP infection is becoming evident from several studies [101,103,104,105]. Increased blood level of osteopontin together with elevated levels of IFN-Î^3^ and IL-17 has been shown possible subclinical biomarkers of bovine MAP infection [103]. Osteopontin provided evidence of inflammatory diseases response as early as 6 h post-infection in a trial of MAP in bovine. Consequently, elevated expression of osteopontin and tumour necrosis factor/transforming growth factor were found as early biomarkers of MAP infection in in vitro macrophages model [103]. However, tumour necrosis factor-a has greater expression in clinical bovine [101,103,104,105]. In another study, osteopontin immune response in the ileocecal lymph node and ileum in MAP infected dairy cows found localized expression of osteopontin in higher frequency in the lamina propria [103].

#### 3.4.10. MAP and Neuromyelitis Optica Spectrum Disorder

MAP antibodies against PknG and PtpA have been found in high levels in neuromyelitis optica spectrum disorder associated with multiple sclerosis patients [70]. However, the aetiological role of MAP in neuromyelitis optica spectrum disorder has not been researched.

### 3.5. Resource-, Intellectual- and Knowledge-Sharing in MAP Research

#### 3.5.1. Country Collaboration Network (CCN) in MAP Research

**Figure 4:**
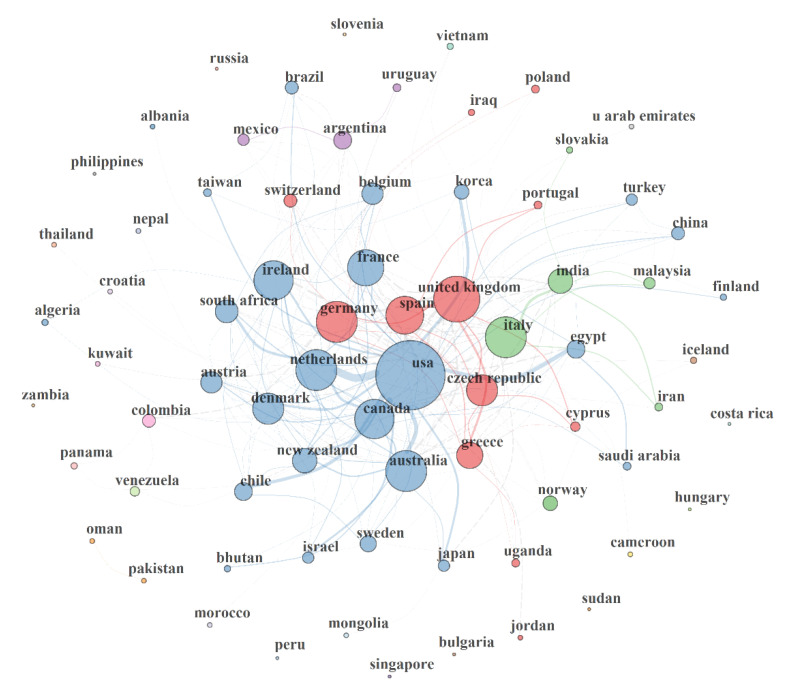
Country collaboration network (CCN) in MAP research from 1911–2019. The collaborative network performance statistics include size = 72; density = 0.099; transitivity = 0.438; diameter = 4; degree of centralization = 0.464; average path length = 2.142; closeness of centralization = 0.027; betweenness of centralization = 0.214; eigenvector of centralization = 0.768; networkDegreeDist = 41. The circle volume of each country correspond to its sharing strength/publication count, connecting curve/line between two countries indicated bidirectional relationship between the countries; the thickness of the curve/line revealed strength of the bidirectional collaborative relationships; countries with the same colour or similar coloured link belong to the same cluster of collaborative community.

This section presents country, institution and author collaboration networks in MAP research from 1911 to 2019. The performance statistics of MAP research in terms of resource-, knowledge and intellectual-networking from the country perspective from 1911–2019 is presented in Figure 4. Four collaboration clusters that consisted in 2 to 17 nations were observed in the network. Intellectual, resources and knowledge flow based on the country collaboration network (CCN, intra-/internationally) possessed characteristics of size (NZ), density (NDE), transitivity (NT), diameter (NDI), degree centralization (NDC), average path length (NAPL), closeness centralization (CC), betweenness centralization (BC), eigenvector centralization (EC), and network-degree-distance (NDD) of 72, 0.099, 0.438, 4, 0.464, 2.142, 0.027, 0.214, 0.768 and 41 respectively. Collaborations tend greatly towards intracontinental countries relationship.

The CCN size suggested that 72 countries participated in MAP research. Also, the CCN density is characteristic of a sparse network. This suggests that the countries have not maximized the possible collaborative relationship that could possibly exist in MAP research. Network density measures the ratio of the number of present collaborative relationships/links given a total number of possible collaborative relationship/links, and is valued from 0 to 1 [106,107]. Generally, the more collaborative ties a cluster of nations/authors/institutions possess, the higher the network density and the faster the collaborative influences and initiative process [106]. A transitivity of 0.438 in CCN denotes a moderate chance (43.8%) of collaborative networking of a country through another country to a third country for knowledge or resources collaboration as this might result from underlying co-occurring competing interest. Transitivity measures the possibility of establishing a connection to a third party through a common connection and is usually influenced by an overlap of competing social interests [107]. However, the CCN diameter (4) suggests a possibility of building strong connections among countries in MAP research. The diameter has a characteristic ‘small world’ network diameter of the human acquaintanceship network (diameter ≤6.0) necessary for positive or negative feedback [108]. Basically, a network diameter defines amount of effort needed or the length of time for information to move from one (country) end to the other in the network [106,107]. The degree centralization of CCN revealed a moderate dominance and influence of some countries (e.g., USA) over the others in MAP research. These countries are probable initiators of collaborative relationship and in most cases provide the central funding or resources supports in their community clusters in the network. Removal of such nations, would lead to fragmentation of the CCN and overall declined on MAP research. Degree centralization points to the pro rata dominance of a single vertex/node (e.g., USA) over all other vertexes/nodes (countries) in the network [107,109]. A centralization of 0 and 1 reveals that all the vertexes have equal centrality (no single node that is more influential than other) and a single vertex is completely dominating other typical vertexes respectively [107,109]. The more connections an individual (nation/author/institution) has in a network, the greater his influence or centrality in the network [106]. Generally, intercontinental collaboration between countries could promote MAP research, especially in Africa, where there is presently little or no research. Considering a great number of ruminant industries, the priority for MAP research in Africa cannot be overemphasized. BC of 0.214 in the CCN revealed that there exist no gatekeeping nation or country for assuring access to other nations. This is further supported by the CC (0.027) which showed that there was little evidence of a dominating country that is directly linked with every other nation in MAP research. The EC of 0.768 suggest that, on average, most nations are connected to other influential nations in MAP.

The appearances of countries such as Cyprus, Jordan, Turkey, Pakistan, Slovenia, Venezuela, Bulgaria, Albania, Algeria, Costa Rica, Indonesia, Iraq, Nepal, Singapore, Thailand, Bhutan, Malaysia, South Africa, Uganda and Kuwait with connections in the CCN was due to studies conducted in other countries, but co-authored by authors from those nations. Such studies were not primarily conducted in the countries. A nation’s productivity in this study was based on corresponding authors’ countries. Otherwise, the countries are probable secondary countries of affiliations of the authors. The USA, Italy (e.g., Italy is connected with Ireland, Iran, Czech Republic, India, France, Australia, Germany, Denmark, Spain, Japan, Switzerland, Greece, Cyprus, Malaysia) and UK (Spain, Greece, Cyprus, Czech Republic, Portugal etc.) played a central role in their respective clusters.

#### 3.5.2. Author Collaboration Network (ACN) in MAP Research

**Figure 5:**
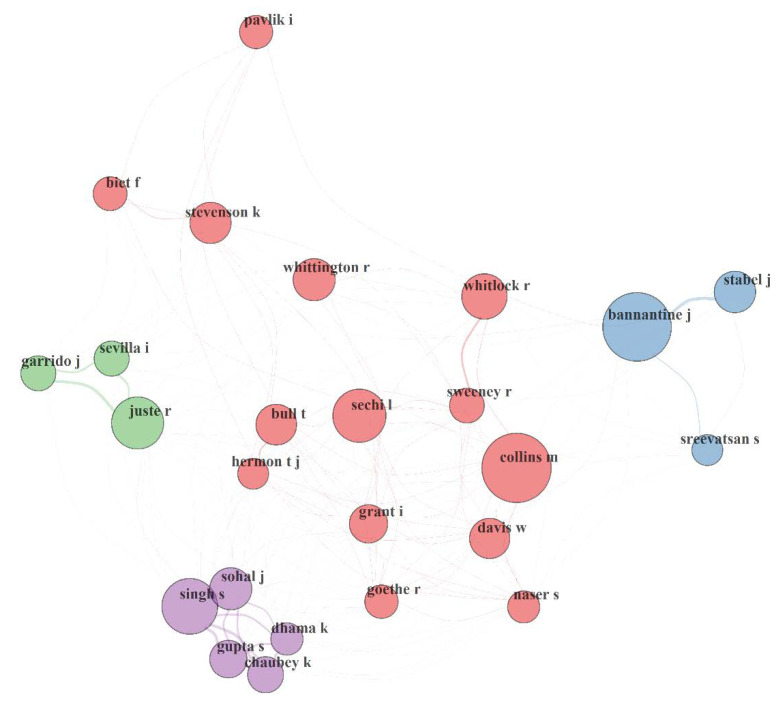
Authors collaboration network (ACN) in MAP research from 1911 to 2019. The collaborative network performance statistics are as listed: size = 6662; density = 0.001; transitivity = 0.387; diameter = 13; degree of centralization = 0.04; average path length = 4.482; closeness of centralization = 0; betweenness of centralization = 0.086; eigenvector of centralization = 0.986; networkDegreeDist = 279. The circle volume of each node (author) correspond to its sharing strength/publication count, connecting curve/line between author nodes indicated bidirectional relationship between the authors; the thickness of the curve/line revealed strength of the bidirectional collaborative/co-author relationships; authors with the same colour or similar coloured link depict that they belong to the same cluster of collaborative/co-authorship community; isolated author(s) or author(s) with no connecting curve with other author(s) is(are) devoid of any collaboration.

Figure 5 presents the authors’ collaboration network (ACN) and its performance statistics from 1911 to 2019. The network possessed 4 clusters of authorship relationship that were made up of at least 3 to 14 individuals. Authors’ collaboration relationships skewed nationally towards their own nation and internationally towards the same continent with few exceptions. This might be indicative of common interests as conditions attached to most funding (e.g., in Europe) demand project/proposal co-sponsored/initiated by institutions/researchers within the same nation/continents (European Union (EU)). Collaboration relationships are globally weak among the 6662 author (size) as suggested by the NDE (0.001). Similarly, there is a low (38.7%) chance of one researcher connecting with another third party scientist via a common connection as shown by the ACN transitivity. Thus, it requires a great deal of effort or time for information to move among the 6662 authors as revealed by the network diameter (13). The diameter doubles the human acquaintanceship network diameter (≤ 6.0) for swift feedback; except for authors who have direct relationships with one another (i.e., diameter = 1). Although, some authors (e.g., Bannantine J; Collins M; Stevenson K) played central roles in their collaboration cluster community, the ACN global NDC of 4% pictured a lack of dominance of one researcher over the others in MAP research overall. The ACN average path length (4.48) reveals that a researcher would need to communicate through or be referred by ≈5 authors/researchers, on average, to establish contacts with a complete unknown/foreign researcher or expert in MAP research domain. The NAPL in a network is defined as the mean (shortest) distance between any couples (all pairs) of its nodes [110]. It simply reflects how quickly collaborative relationships could be initiated with an unknown expert by referral chains in a knowledge domain.

#### 3.5.3. Institution Collaboration Network (ICN) in MAP Research

**Figure 6:**
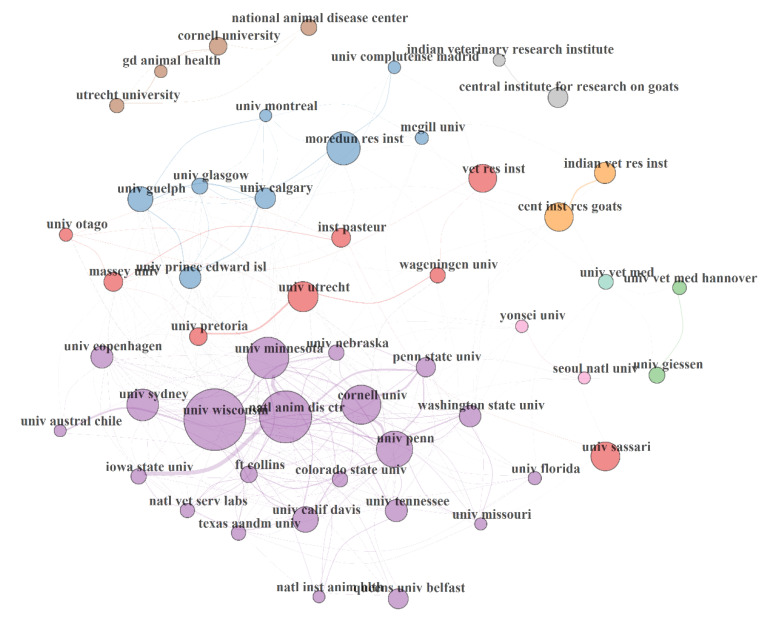
**Institution collaboration network (ICN) in MAP research from 1911 to 2019.** The top 50 institutions were displayed. The collaborative network performance statistics are as listed: size = 2124, density = 0.002, transitivity = 0.235; diameter = 15; degree of centralization = 0.049; average path length = 5.408, closeness of centralization = 0.001, betweenness of centralization = 0.088, eigenvector of centralization = 0.98 and networkDegreeDist = 279. The circle volume of each institution correspond to its sharing strength/publication count, connecting curve/line between two institutions indicated bidirectional relationship between the institutions; the thickness of the curve/line revealed strength of the bidirectional collaborative relationships; institutions with the same colour belong to the same cluster of collaborative institution community; isolated institution(s) is/are deficient in collaboration.

The institution collaboration network (ICN) in MAP research from 1911 to 2019 is shown in Figure 6. The network statistics are as follow: size = 2124, density = 0.002, transitivity = 0.235; diameter = 15; NDC = 0.049; NAPL = 5.408, CC = 0.001, BC = 0.088, EC = 0.98 and NDD = 279. ICN had four clusters of collaborative institution (with the lowest and largest comprised of 3 and 22 institutions respectively) in MAP during the period. The majority of the network statistics (density, transitivity, NDC, CC, BC) pointed out poor collaboration and acquaintanceship among 2124 institutions that were involved in MAP. In fact, this is reflective of national and continental skewness or orientation of MAP research. Collaborations also tend to be intracontinental institutional phenomena. Meanwhile, inter-institutional collaborative relationships could advance MAP research in institutions with low resources or that lack equipment/technical expertise required for MAP research. However, the EC of 0.98 revealed that authors are connected to other influential researchers in MAP research domain.

## 4. Conclusions

The bibliometric and growth analytic review of MAP from 1911–2019 found a yearly growth rate of 6.31% and positive growth in relation to type 1 diabetes mellitus, rheumatoid arthritis, food science and technology, immunology, agriculture, pathology, and research and experimental medicine, wildlife, environments, virulence, disease resistance, meat and meat products, osteopontin, waste milk and digester slurry/sludge subjects; negative growth in ulcerative colitis and Parkinson’s disease; but no growth in multiple sclerosis, sarcoidosis, thyroid disorders, psoriasis, and lupus. Collaborations tend to be intracontinental phenomena and MAP research presently is limited to North America, Europe, Asia, South America, Oceania, Turkey and a few nations in Africa. In conclusion, inadequate resources-, knowledge- and scientific-networking hampered growth and awareness of MAP research globally. The study, therefore, recommends further research to strengthen or match evidence of MAP’s epidemiologic prevalence in autoimmune diseases and proffer practical solutions in drug development and point-of-care diagnostics amongst other extended themes.

## Figures and Tables

**Table 1:** Types and characteristics of MAP research from 1911–2019.

Attributes	Counts/Rates
Documents	3889
Sources	581
Keywords Plus	5175
Author’s Keywords	3110
Average citations/documents	20.65
Authors	6662
Author Appearances	18,666
Authors of single-authored documents	217
Authors of multi-authored documents	6445
Single-authored documents	348
Documents/ Author	0.584
Authors/Document	1.71
Co-Authors/Documents	4.8
Collaboration Index	1.82
Document types	
Article	3737
Article, book chapter	38
Article, early access	3
Article, proceedings paper	111

**Table 2:** The top 20 productive/active authors in MAP research from 1911–2019.

R	Authors	Articles	% of 3889	AGR	ADY	PDLY	h-Index
1	Whittington, R.J.	143	3.68	0	4.3	10.8	34
2	Collins, M.T.	130	3.34	30	1.7	5.6	35
3	Stabel, J.R.	125	3.21	−100	2.7	8.3	30
4	Singh, S.V.	111	2.85	−170	6	27.7	26
5	Bannantine, J.P.	109	2.80	30	4.7	14.4	29
6	Whitlock, R.H.	84	2.16	−30	0	0	28
7	Sohal, J.S.	71	1.83	−80	2.7	17.4	25
8	Juste, R.A.	64	1.65	−30	2.3	14.3	21
9	Nielsen, S.S.	64	1.65	−130	0.7	3.5	23
10	Pavlik, I.	60	1.54	0	0	0	22
11	Sechi, L.A.	59	1.52	−170	3.3	18.9	19
12	Singh, P.K.	58	1.49	0	0	0	25
13	Stevenson, K.	57	1.47	−100	1	6.5	24
14	Singh, A.V.	55	1.41	0	0	0	25
15	Barkema, H.W.	54	1.39	−200	3.3	20.4	19
16	Begg, D.J.	54	1.39	0	3	20.9	15
17	De Buck, J.	52	1.34	−200	3	22	15
18	Koets, A.P.	51	1.31	0	0.3	3.7	15
19	Garrido, J.M.	50	1.29	30	2	14.3	19
20	Grant, I.R.	49	1.26	−70	1.7	12.2	27
21	Schukken, Y.H.	49	1.26	−100	1.7	12.8	19
22	Wells, S.J.	49	1.26	30	1	7.7	21

R = rank based on total number of articles; average growth rate (AGR); average documents per year (ADY); percentage document per last 3 years (PDLY).

**Table 3:** The top cited titles in MAP research from 1911 to 2019.

S/n	Author	Paper	Reference	TC	TCperYear
1	Ott et al.	“Herd-level economic losses associated with Johne’s disease on US dairy operations”	[6]	439	20.9
2	Naser et al.	“Culture of *Mycobacterium avium* subspecies *Para tuberculosis* from the blood of patients with Crohn’s disease”	[31]	434	27.12
3	Green et al.	“Sequence and characteristics or IS 900, an insertion element identified in a human Crohn’s disease isolate or Mycobacterium Para tuberculosis”	[32]	370	11.94
4	Sanderson et al.	“Mycobacterium Para tuberculosis DNA in Crohn’s disease tissue”	[33]	322	11.5
5	Li et al.	“The complete genome sequence of Mycobacterium avium subspecies Para tuberculosis”	[34]	316	21.07
6	Sweeney et al.	“Mycobacterium Para tuberculosis cultured from milk and supramammary lymph nodes of infected asymptomatic cows”	[35]	315	11.25
7	Bull et al.	“Detection and verification of Mycobacterium avium subsp. Para tuberculosis in fresh ileocolonic mucosal biopsy specimens from individuals with and without Crohn’s disease”	[29]	307	18.06
8	Vary et al.	“Use of highly specific DNA probes and the polymerase chain reaction to detect Mycobacterium Para tuberculosis in Johne’s disease”	[36]	305	10.17
9	Sweeney et al.	“Mycobacterium Para tuberculosis isolated from fetuses of infected cows not manifesting signs of the disease”	[37]	299	10.68

R = rank based on TC (total citations).

**Table 4:** Country participations in MAP research from 1911–2019.

R	Country	Articles	% of 3889	Freq (%)	SCA	SCA% of 3889	SCA/Articles (%)	MCA	% of 3889	MCA/Articles (%)
1	USA	887	22.81	26.20	726	18.67	81.85	161	4.14	18.15
2	Australia	236	6.07	6.97	209	5.37	88.56	27	0.69	11.44
3	India	204	5.25	6.02	180	4.63	88.24	24	0.62	11.77
4	Canada	199	5.12	5.88	159	4.09	79.90	40	1.03	20.10
5	United Kingdom	192	4.94	5.67	148	3.81	77.08	44	1.13	22.92
6	Germany	183	4.71	5.40	155	3.99	84.70	28	0.72	15.30
7	Italy	125	3.21	3.69	91	2.34	72.80	34	0.87	27.2
8	Spain	113	2.91	3.34	88	2.26	77.88	25	0.64	22.12
9	Ireland	105	2.70	3.10	80	2.06	76.19	25	0.64	23.81
10	New Zealand	94	2.42	2.78	74	1.90	78.72	20	0.51	21.28
11	Netherlands	91	2.34	2.69	64	1.65	70.33	27	0.69	29.67
12	Denmark	82	2.11	2.42	72	1.85	87.80	10	0.26	12.20
13	France	73	1.88	2.16	55	1.41	75.34	18	0.46	24.66
14	Brazil	66	1.70	1.95	62	1.59	93.94	4	0.10	6.06
15	Czech Republic	61	1.57	1.80	49	1.26	80.33	12	0.31	19.67
16	Iran	50	1.29	1.48	44	1.13	88.00	6	0.15	12.00
17	Japan	50	1.29	1.48	36	0.93	72.00	14	0.36	28.00
18	Norway	44	1.13	1.30	38	0.98	86.36	6	0.15	13.64
19	Austria	43	1.11	1.27	32	0.82	74.42	11	0.28	25.58
20	Chile	43	1.11	1.27	22	0.57	51.16	21	0.54	48.84
21	Belgium	41	1.05	1.21	35	0.90	85.37	6	0.15	14.63
22	Argentina	40	1.03	1.18	34	0.87	85.00	6	0.15	15.00
23	Korea	38	0.98	1.12	32	0.82	84.21	6	0.15	15.79
24	Switzerland	26	0.67	0.77	19	0.49	73.08	7	0.18	26.92
25	Turkey	26	0.67	0.77	26	0.67	100.00	0	0.00	0
26	Greece	25	0.64	0.74	15	0.39	60.00	10	0.26	40.00
27	Poland	25	0.64	0.74	23	0.59	92.00	2	0.05	8.00
28	Portugal	21	0.54	0.62	16	0.41	76.19	5	0.13	23.81
29	Colombia	19	0.49	0.56	15	0.39	78.95	4	0.10	21.05
30	Hungary	19	0.49	0.56	14	0.36	73.68	5	0.13	26.32
31	Mexico	19	0.49	0.56	17	0.44	89.47	2	0.05	10.53
32	Saudi Arabia	19	0.49	0.56	14	0.36	73.68	5	0.13	26.32
33	Sweden	16	0.41	0.47	10	0.26	62.50	6	0.15	37.5
34	China	15	0.39	0.44	13	0.33	86.67	2	0.05	13.33
35	Pakistan	11	0.28	0.32	11	0.28	100.00	0	0.00	0
36	Egypt	9	0.23	0.27	8	0.21	88.89	1	0.03	11.11
37	Israel	9	0.23	0.27	4	0.10	44.44	5	0.13	55.56
38	Iceland	6	0.15	0.18	5	0.13	83.33	1	0.03	16.67
39	Jordan	6	0.15	0.18	5	0.13	83.33	1	0.03	16.67
40	Slovakia	6	0.15	0.18	4	0.10	66.67	2	0.05	33.33
41	Slovenia	4	0.10	0.12	4	0.10	100.00	0	0.00	0
42	South Africa	4	0.10	0.12	4	0.10	100.00	0	0.00	0
43	Sudan	4	0.10	0.12	4	0.10	100.00	0	0.00	0
44	Uganda	3	0.08	0.09	1	0.03	33.33	2	0.05	66.67
45	Venezuela	3	0.08	0.09	3	0.08	100.00	0	0.00	0
46	Bulgaria	2	0.05	0.06	2	0.05	100.00	0	0.00	0
47	Morocco	2	0.05	0.06	1	0.03	50.00	1	0.03	50.00
48	Peru	2	0.05	0.06	2	0.05	100.00	0	0.00	0
49	Philippines	2	0.05	0.06	2	0.05	100.00	0	0.00	0
50	Taiwan	2	0.05	0.06	1	0.03	50.00	1	0.03	50.00
51	Albania	1	0.03	0.03	1	0.03	100.00	0	0.00	0
52	Algeria	1	0.03	0.03	1	0.03	100.00	0	0.00	0
53	Bhutan	1	0.03	0.03	0	0.00	0.00	1	0.03	100.00
54	Cameroon	1	0.03	0.03	0	0.00	0.00	1	0.03	100.00
55	Costa Rica	1	0.03	0.03	1	0.03	100.00	0	0.00	0
56	Croatia	1	0.03	0.03	0	0.00	0.00	1	0.03	100.00
57	Cyprus	1	0.03	0.03	0	0.00	0.00	1	0.03	100.00
58	Finland	1	0.03	0.03	0	0.00	0.00	1	0.03	100.00
59	Ghana	1	0.03	0.03	1	0.03	100.00	0	0.00	0
60	Indonesia	1	0.03	0.03	1	0.03	100.00	0	0.00	0
61	Iraq	1	0.03	0.03	1	0.03	100.00	0	0.00	0
62	Kuwait	1	0.03	0.03	1	0.03	100.00	0	0.00	0
63	Nepal	1	0.03	0.03	1	0.03	100.00	0	0.00	0
64	Oman	1	0.03	0.03	0	0.00	0.00	1	0.03	100.00
65	Panama	1	0.03	0.03	0	0.00	0.00	1	0.03	100.00
66	Russia	1	0.03	0.03	1	0.03	100.00	0	0.00	0
67	Singapore	1	0.03	0.03	1	0.03	100.00	0	0.00	0
68	Thailand	1	0.03	0.03	1	0.03	100.00	0	0.00	0
69	U Arab Emirates	1	0.03	0.03	0	0.00	0.00	1	0.03	100.00
70	Uruguay	1	0.03	0.03	0	0.00	0.00	1	0.03	100.00

R = rank in terms of T, T = total articles, Freq = frequency of publication; SCA = single country articles; SCA/T = single country articles-ratio; MCA = multiple country articles; MCA/T = multiple country articles-ratio.

**Table 5:** Top 20 important journal outlets on MAP research from 1911–2019.

R	Sources	Articles	% of 3889	AGR	ADY	PDLY	h-Index	IJCR(SJR)
1	Veterinary Microbiology	186	4.78	−70	2	3.5	44	2.791(1.17)
2	Journal of Dairy Science	162	4.17	0	13.3	25.3	33	
3	Preventive Veterinary Medicine	147	3.78	−100	4.3	9.5	34	2.302(1.1)
4	Veterinary Immunology and Immunopathology	137	3.52	−30	4	10.3	25	
5	American Journal of Veterinary Research	133	3.42	0	0	0	30	1.070(0.63)
6	Journal of Clinical Microbiology	110	2.83	−30	0	0	49	4.959(2.31)
7	Journal of Veterinary Diagnostic Investigation	85	2.19	0	1.7	6.3	25	1.174(0.6)
8	Australian Veterinary Journal	82	2.11	−30	0	0	25	0.887(0.41)
9	Applied and Environmental Microbiology	72	1.85	−70	0	0	38	4.077(1.66)
10	Infection and Immunity	72	1.85	0	0.7	3	35	3.160(1.59)
11	Plos One	68	1.75	−300	4.3	19.1	22	2.776(1.1)
12	Journal of The American Veterinary Medical Association	62	1.59	0	0	0	30	
13	Veterinary Record	59	1.52	70	2.3	11.7	18	
14	Veterinary Research	52	1.34	−100	2.7	19.5	15	
15	Journal of Comparative Pathology	43	1.11	0	0	0	17	
16	Clinical and Vaccine Immunology	41	1.05	0	0.7	5.1	17	3.233(1.4)
17	Berliner Und Munchener Tierarztliche Wochenschrift	39	1.00	−30	2.7	27.6	8	
18	Small Ruminant Research	36	0.93	0	1.3	12.1	11	
19	Pesquisa Veterinaria Brasileira	34	0.87	−30	0.7	9.5	8	
20	Acta Veterinaria Scandinavica	33	0.85	0	0	0	11	

R = rank based on total articles; TC= total citation; IJCR(SJR) = InCite Journal Citation Reports (Scientific Journal Rankings); C = country where publisher is based; PU = publishing house; average growth rate (AGR); average documents per year (ADY); percentage document per last 3 years (PDLY).

**Table 6:** Trend and growth of MAP research-based research topics from 1911–2019.

Topic	No. Articles	% of 3889	AGR	ADY	PDLY	h-Index
Livestock	634	16.30	−30.00	27	12.8	51
Milk	203	5.22	−130.00	12.7	18.7	35
Dairy	154	3.96	0.00	8.3	16.2	28
Feces	57	1.47	−70.00	2.3	12.3	18
Antibody	43	1.11	0.00	2	14	15
Wildlife	43	1.11	30.00	1.3	9.3	13
Environments	32	0.82	100.00	3.7	34.4	12
Virulence	14	0.36	70.00	1.3	28.6	7
Water	12	0.31	−30.00	1	25	6
Resistance	8	0.21	100.00	1	37.5	5
Meat and meat products	6	0.15	30.00	0.3	16.7	5
Wastewaters	4	0.10	30.00	0.7	50	2
Feed	3	0.08	0.00	0.3	33.3	2
Microbiota	3	0.08	−30.00	0	0	3
Paediatric	3	0.08	0.00	0	0	3
Anaerobic digestion	2	0.05	30.00	0.7	100	1
Manure	0	0.00	0.00	0	0	0
Pasture/pasteurisation	0	0.00	0.00	0	0	0
Sewage	0	0.00	0.00	0	0	0
Urine	0	0.00	0.00	0	0	0

Average growth rate (AGR); average documents per year (ADY); percentage documents per last 3 years (PDLY).

**Table 7:** Growth of MAP research related to autoimmune disorders.

Disorder	Total	AGR	ADY	PDLY	h-Index
Ulcerative colitis	13	−30	0	0	9
Multiple sclerosis	10	0	0.7	20	6
Resistance (disease)	8	**100**	1	37.5	5
Type 1 diabetes	6	**70**	1	50	4
Osteopontin	5	**70**	0.7	40	2
Rheumatoid arthritis	4	**100**	1.3	100	1
Sarcoidosis	2	0	0.3	50	1
Thyroid disorders	2	0	0.7	100	2
Parkinson’s disease	1	−30	0	0	1
transthyretin	1	0	0	0	1
Psoriasis	0	0	0	0	0
Lupus	0	0	0	0	0

Average growth rate (AGR); average documents per year (ADY); percentage document per last 3 years (PDLY).

## Supplementary Materials

The following are available online at https://www.mdpi.com/2076-2607/8/8/1212/s1: Table S1: MAP research total citations per country, Table S2: Growth of MAP research in relation to 52 scientific subject areas., Table S3: MAP research growth by country from 2017–2019, Table S4: Growth of MAP research based on institution participations in the last 3 years.
