# Supplementary material for: Systematic Assessment of Mycobacterium avium Subspecies Paratuberculosis Infections from 1911–2019: A Growth Analysis of Association with Human Autoimmune Diseases
Source: Microorganisms. 2020 Aug 10;8(8):1212. doi: 10.3390/microorganisms8081212 (PMC7465227; doi:10.3390/microorganisms8081212)
Supplement: Supplementary file 1 [file microorganisms-08-01212-s001.pdf]

Table S1: MAP research total citations per country.

Rank	Country	TCitations	AACitations
1	USA	26418	29.78
2	United Kingdom	7723	40.22
3	Australia	6014	25.48
4	Canada	3875	19.47
5	Ireland	2942	28.02
6	Spain	2914	25.79
7	Netherlands	2767	30.41
8	Germany	2373	12.97
9	Denmark	2344	28.59
10	New Zealand	2205	23.46
11	France	1680	23.01
12	Czech Republic	1679	27.52
13	Italy	1634	13.07
14	India	1328	6.51
15	Belgium	1039	25.34
16	Norway	1004	22.82
17	Japan	839	16.78
18	Switzerland	517	19.88
19	Brazil	439	6.65
20	Sweden	436	27.25

TC (total citations); AAC (average article citations); Average Growth Rate (AGR); Average Documents per Year (ADY); percentage document per last 3 years (PDLY)

Table S2: Growth of MAP research in relation to 52 scientific subject areas.

R	subject	articles	% of 3889	AGR	ADY	PDLY	h-index
1	Veterinary Sciences	1450	37.28	-370	48	9.9	67
2	Microbiology	702	18.05	-230	20	8.5	72
3	Immunology	373	9.59	**30**	13.3	10.7	45
4	Agriculture	336	8.64	**30**	25.7	22.9	35
5	Food Science & Technology	234	6.02	**70**	16.7	21.4	40
6	Infectious Diseases	181	4.65	-170	4	6.6	40
7	Biotechnology & Applied Microbiology	175	4.50	-170	7	12	45
8	Biochemistry & Molecular Biology	92	2.37	-70	5.3	17.4	25
9	Science & Technology - Other Topics	90	2.31	-300	8.3	27.8	23
10	Gastroenterology & Hepatology	73	1.88	-30	1.3	5.5	33
11	Pathology	52	1.34	**30**	0.7	3.8	25
12	Research & Experimental Medicine	41	1.05	**30**	1.3	9.8	18
13	Genetics & Heredity	37	0.95	0	2	16.2	16
14	Cell Biology	27	0.69	-30	0.7	7.4	17
15	Public, Environmental & Occupational Health	21	0.54	-130	0.3	4.8	12
16	Life Sciences & Biomedicine - Other Topics	19	0.49	0	1.3	21.1	8
17	Zoology	14	0.36	-30	0.7	14.3	5
18	Chemistry	13	0.33	0	0.7	15.4	6
19	Neurosciences & Neurology	12	0.31	-70	1	25	6
20	General & Internal Medicine	8	0.21	-30	0.7	25	4
21	Mathematical & Computational Biology	8	0.21	0	0.7	25	5
22	Pharmacology & Pharmacy	8	0.21	0	0.3	12.5	6
23	Reproductive Biology	7	0.18	0	0.3	14.3	4
24	Engineering	5	0.13	0	0.3	20	4
25	Environmental Sciences & Ecology	5	0.13	0	0	0	4
26	Respiratory System	5	0.13	-30	0.7	40	3
27	Hematology	4	0.10	0	0	0	4
28	Biophysics	3	0.08	0	0.3	33.3	2
29	Entomology	3	0.08	0	0	0	3
30	Instruments & Instrumentation	3	0.08	0	0.3	33.3	2
31	Medical Laboratory Technology	3	0.08	0	0	0	2
32	Business & Economics	2	0.05	0	0	0	2
33	Mathematics	2	0.05	0	0	0	2
34	Parasitology	2	0.05	0	0.3	50	0
35	Tropical Medicine	2	0.05	0	0.3	50	0
36	Virology	2	0.05	0	0	0	1
37	Water Resources	2	0.05	0	0	0	2
38	Biodiversity & Conservation	1	0.03	0	0	0	1
39	Electrochemistry	1	0.03	0	0.3	100	1
40	Evolutionary Biology	1	0.03	0	0	0	1
41	Fisheries	1	0.03	0	0	0	1

42	Mathematical Methods In Social Sciences	1	0.03	0	0	0	1
43	Nuclear Science & Technology	1	0.03	0	0	0	1
44	Nutrition & Dietetics	1	0.03	0	0	0	1
45	Oncology	1	0.03	0	0.3	100	1
46	Paediatrics	1	0.03	0	0	0	1
47	Physics	1	0.03	0	0	0	1
48	Physiology	1	0.03	0	0	0	1
49	Rheumatology	1	0.03	0	0.3	100	1
50	Spectroscopy	1	0.03	0	0	0	1
51	Surgery	1	0.03	0	0	0	1
52	Toxicology	1	0.03	0	0	0	1

Average Growth Rate (AGR); Average Documents per Year (ADY); percentage document per last 3 years (PDLY)

Table S3: MAP research growth by country from 2017 – 2019.

Country	Articles	% of 3889	AGR%	ADY	PDLY	h-index
United States	965	24.81	**30**	26	8.1	71
Australia	267	6.87	0	9	10.1	45
United Kingdom	240	6.17	-330	8	10	48
India	228	5.86	-430	13	17.1	25
Canada	223	5.73	-330	13	17.5	36
Germany	180	4.63	**170**	10.7	17.8	26
Italy	139	3.57	-170	10.3	22.3	23
Netherlands	136	3.50	-70	4.3	9.6	41
Spain	129	3.32	**100**	6.7	15.5	34
New Zealand	106	2.73	0	2.3	6.6	22
Denmark	90	2.31	-130	1.7	5.6	30
France	72	1.85	-30	3.7	15.3	19
Czech Republic	71	1.83	-30	1.3	5.6	23
Ireland	66	1.70	0	5.7	25.8	16
North Ireland	63	1.62	-70	3.3	15.9	28
Iran	60	1.54	-30	5.3	26.7	7
Brazil	57	1.47	-30	3.7	19.3	12
South Korea	51	1.31	-30	4	23.5	12
Japan	46	1.18	-100	1.3	8.7	16
Argentina	45	1.16	-100	1.3	8.9	10
Egypt	45	1.16	**170**	5	33.3	11
Norway	43	1.11	0	0	0	18
Austria	39	1.00	0	2.7	20.5	11
Belgium	37	0.95	0	1	8.1	19
Chile	37	0.95	**70**	2.7	21.6	13
Greece	34	0.87	-70	0.7	5.9	13

Switzerland	25	0.64	**30**	0.7	8	11
Turkey	25	0.64	-30	1.7	20	6
Portugal	22	0.57	-30	1.7	22.7	7
South Africa	22	0.57	0	0.7	9.1	13
Poland	21	0.54	**30**	1	14.3	3
Saudi Arabia	21	0.54	**70**	1	14.3	7
Colombia	19	0.49	0	1.7	26.3	6
Mexico	19	0.49	-30	0.7	10.5	6
Sweden	19	0.49	0	0	0	14
China	18	0.46	0	2.7	44.4	7
Hungary	16	0.41	0	0.3	6.2	5
Israel	15	0.39	0	0.3	6.7	8
Pakistan	13	0.33	0	1.7	38.5	3
Taiwan	9	0.23	0	0	0	7
Jordan	8	0.21	0	0	0	5
Slovakia	8	0.21	0	0	0	6
Cyprus	7	0.18	-30	0.3	14.3	6
Iceland	6	0.15	0	0	0	4
Malaysia	6	0.15	0	0.3	16.7	4
Bhutan	5	0.13	0	0.7	40	4
Venezuela	5	0.13	0	0	0	2
Finland	4	0.10	0	0	0	4
Sudan	4	0.10	0	0	0	2
Thailand	4	0.10	**70**	1	75	1
Uganda	4	0.10	0	0.3	25	3
Uruguay	4	0.10	**70**	1	75	2
Slovenia	3	0.08	0	0	0	2
Albania	2	0.05	0	0	0	0
Algeria	2	0.05	**30**	0.3	50	1
Bulgaria	2	0.05	**70**	0.7	100	0
Croatia	2	0.05	0	0	0	0
Iraq	2	0.05	0	0.3	50	1
Kuwait	2	0.05	-70	0	0	1
Peru	2	0.05	0	0	0	1
Philippines	2	0.05	0	0.7	100	1

Average Growth Rate (AGR); Average Documents per Year (ADY); percentage document per last 3 years (PDLY)

Table S4: Growth of MAP research based on institutional participations in the last 3 years.

S/n	Institution with country	Total	% of 3889	AGR	ADY	PDLY	h-index
1	Univ Sydney, Australia	85	2.19	**30**	5.3	18.8	21
2	Coll Vet Med, United States	78	2.01	0	0	0	34
3	Univ Wisconsin, United States	78	2.01	-30	3.3	12.8	20
4	Sch Vet Med, United States	76	1.95	0	0	0	34
5	USDA ARS, United States	63	1.62	0	4	19	18
6	Univ Minnesota, United States	61	1.57	**30**	1.7	8.2	20
7	Cornell Univ, United States	60	1.54	-70	3.3	16.7	22
8	Univ Calgary, Canada	51	1.31	-230	4	23.5	17
9	Univ Guelph, Canada	48	1.23	-130	5.7	35.4	15
10	Natl Anim Dis Ctr, United States	47	1.21	0	0	0	28
11	ARS, United States	46	1.18	0	2.7	17.4	18
12	Cent Inst Res Goats, India	46	1.18	-200	3.7	23.9	13
13	Univ Sassari, Italy	46	1.18	-200	3.7	23.9	14
14	Univ Penn, United States	39	1.00	-100	0.3	2.6	20
15	Vet Res Inst, Czech Republic	38	0.98	-30	1.3	10.5	14
16	Univ Copenhagen, Denmark	36	0.93	-130	0.7	5.6	16
17	Iowa State Univ, United States	33	0.85	-70	1.3	12.1	11
18	Moredun Res Inst, United Kingdom	33	0.85	-100	1.3	12.1	16
19	Univ Utrecht, Netherlands	33	0.85	-30	0.3	3	18
20	Indian Vet Res Inst, India	32	0.82	-230	2.7	25	6
21	Fac Vet Sci, Australia	31	0.80	0	0	0	19
22	NEIKER Tecnalia, Spain	29	0.75	-70	1	10.3	13
23	Elizabeth Macarthur Agr Inst, Australia	28	0.72	0	0	0	19
24	Univ Giessen, Germany	25	0.64	-100	0	0	10
25	Washington State Univ, United States	25	0.64	**30**	2	24	14
26	Penn State Univ, United States	24	0.62	0	1.7	20.8	11
27	Michigan State Univ, United States	23	0.59	**30**	1.7	21.7	11
28	Univ Austral Chile, Chile	23	0.59	0	2	26.1	8
29	Massey Univ, New Zealand	21	0.54	-30	0.7	9.5	10
30	Texas A&M Univ, United States	21	0.54	-30	0	0	7
31	Univ Coll Dublin, Ireland	21	0.54	**30**	2.7	38.1	10
32	Univ Otago, New Zealand	21	0.54	0	0.7	9.5	10
33	Univ Prince Edward Isl, Canada	21	0.54	-30	1.7	23.8	11
34	Dept Anim Sci, United States	20	0.51	0	0	0	15
35	Univ Calif Davis, United States	20	0.51	**30**	1	15	9
36	Univ Tennessee, United States	20	0.51	-70	0.7	10	11
37	Queens Univ Belfast, North Ireland	19	0.49	-30	2	31.6	11
38	Univ Pretoria, South Africa	19	0.49	0	0.3	5.3	12
39	Dept Microbiol, United States	18	0.46	0	0	0	16
40	INRA, France	18	0.46	0	1	16.7	10
41	New Bolton Ctr, United States	18	0.46	0	0	0	15
42	Shiraz Univ, Iran	18	0.46	**70**	3.7	61.1	4
43	Tech Univ Denmark, Denmark	18	0.46	-70	1	16.7	9

44	Univ Vet Med Hannover, Germany	18	0.46	0	1	16.7	7
45	AgResearch, New Zealand	17	0.44	**30**	1	17.6	10
46	CS-62132 Brno, Czech Republic	17	0.44	0	0	0	13
47	Cairo Univ, Egypt	17	0.44	0	1	17.6	7
48	Friedrich Loeffler Inst, Germany	17	0.44	-30	1.3	23.5	8
49	USDA, United States	17	0.44	-30	0	0	11
50	Atlantic Vet Coll, Canada	16	0.41	0	0	0	14
51	Fac Vet, Spain	16	0.41	0	0	0	14
52	GLA Univ, India	15	0.39	-30	3.3	66.7	3
53	Orange, Australia	15	0.39	0	0	0	13
54	Wageningen Univ, Netherlands	15	0.39	**70**	1.7	33.3	8
55	NL-7400 AA Deventer, Netherlands	14	0.36	0	0	0	11
56	Univ Florida, United States	14	0.36	**30**	0.3	7.1	8
57	Univ Montreal, Canada	14	0.36	0	2	42.9	6
58	Univ Pisa, Italy	14	0.36	0	2.7	57.1	5
59	Amity Univ Rajasthan, India	13	0.33	-130	1.7	38.5	4
60	Sch Med, United Kingdom	13	0.33	0	0	0	11
61	Seoul Natl Univ, South Korea	13	0.33	-70	1.7	38.5	6
62	TEAGASC, Ireland	13	0.33	**30**	1.7	38.5	5
63	Univ Fed Vicosa, Brazil	13	0.33	-70	1	23.1	5
64	Univ Nebraska, United States	13	0.33	0	0.3	7.7	8
65	Univ Nottingham, United Kingdom	13	0.33	-170	1	23.1	7
66	Anim Dis Diagnost Lab, United States	12	0.31	0	0	0	10
67	Biomed Genom Ctr, United States	12	0.31	0	0	0	11
68	Cork Inst Technol, Ireland	12	0.31	-30	1	25	7
69	Dept Med, United States	12	0.31	0	0	0	11
70	Dept Vet Pathobiol, United States	12	0.31	0	0	0	12
71	Fac Vet Med, Netherlands	12	0.31	0	0	0	10
72	Univ Cagliari, Italy	12	0.31	-100	0	0	9
73	Univ Leon, Spain	12	0.31	-30	1	25	7
74	Anim Hlth Ireland, Ireland	11	0.28	**70**	2.3	63.6	4
75	Colorado State Univ, United States	11	0.28	0	0	0	7
76	Dept Food Sci Food Microbiol, North Ireland	11	0.28	0	0	0	11
77	Dept Pathobiol Sci, United States	11	0.28	0	0	0	10
78	Dept Vet & Biomed Sci, United States	11	0.28	0	0	0	9
79	Fed Res Inst Anim Hlth, Germany	11	0.28	-30	1	27.3	8
80	GD Anim Hlth Serv, Netherlands	11	0.28	-30	1	27.3	5
81	Inst Mikrobiol & Tierseuchen, Germany	11	0.28	0	0	0	7
82	Ist Zooprofilatt Sperimentale Lombardia & Emilia, Italy	11	0.28	**30**	1.3	36.4	5
83	Univ Antioquia, Colombia	11	0.28	**30**	1	27.3	6
84	Univ Complutense Madrid, Spain	11	0.28	**30**	1	27.3	6
85	Univ Glasgow, United Kingdom	11	0.28	-100	1	27.3	5
86	Univ Missouri, United States	11	0.28	-30	0.7	18.2	8
87	Univ Thessaly, Greece	11	0.28	-70	0.3	9.1	6
88	Coll Vet Med & Biomed Sci, United States	10	0.26	0	0	0	9

89	Dept Anim Sci & Anim Hlth, Denmark	10	0.26	0	0	0	9
90	Dept Mol Biol & Microbiol, United States	10	0.26	0	0	0	9
91	Div Pathol, India	10	0.26	0	0	0	8
92	King Faisal Univ, Saudi Arabia	10	0.26	30	0.7	20	4
93	Natl Inst Anim Hlth, Japan	10	0.26	-70	0.3	10	6
94	SERIDA, Spain	10	0.26	70	1.3	40	6
95	Thuringian Anim Dis Fund, Germany	10	0.26	-100	1	30	6
96	Univ Illinois, United States	10	0.26	-100	1.7	50	6
97	Univ Saskatchewan, Canada	10	0.26	-70	0.3	10	8
98	Western Coll Vet Med, Canada	10	0.26	0	0	0	8
99	N-0033 Oslo, Norway	9	0.23	0	0	0	7

Average Growth Rate (AGR); Average Documents per Year (ADY); percentage document per last 3 years (PDLY).
